# Supplementary material for: Attribution of credit in acknowledgements: The case of systematic reviews in medicine
Source: PLoS One. 2026 Jan 6;21(1):e0338714. doi: 10.1371/journal.pone.0338714 (PMC12773806; doi:10.1371/journal.pone.0338714)
Supplement: S1 File — (DOCX) [file pone.0338714.s001.docx]

**S1 File. Supplemental figures.**


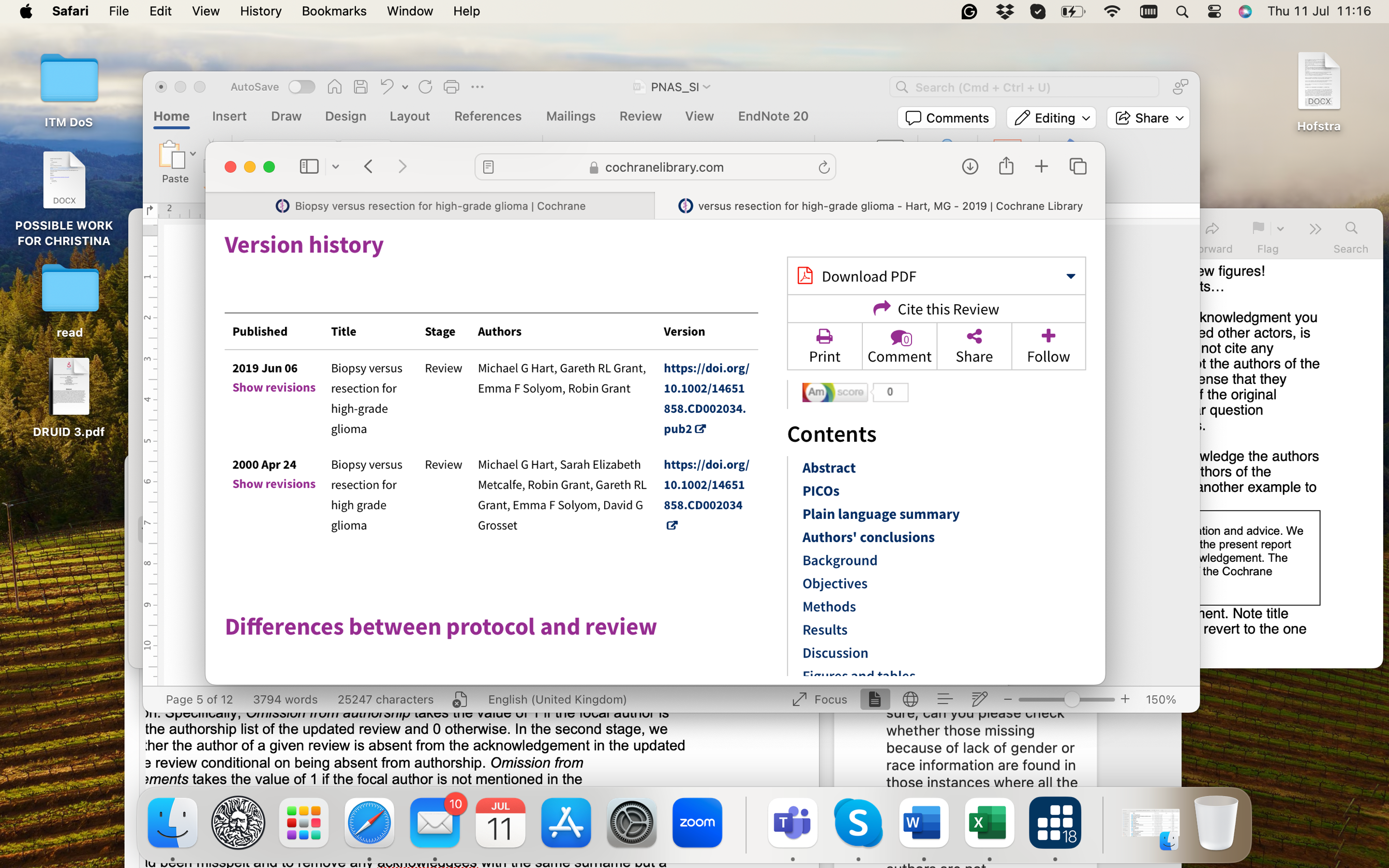


**Fig S1A. Version history information.**

We considered the review “[Biopsy versus resection for high-grade glioma](https://www.cochranelibrary.com/cdsr/doi/10.1002/14651858.CD002034.pub2/information#versionTable)”, Accessed 01 June 2025.

**Fig S1B. Flowchart of the sample construction process.**

**
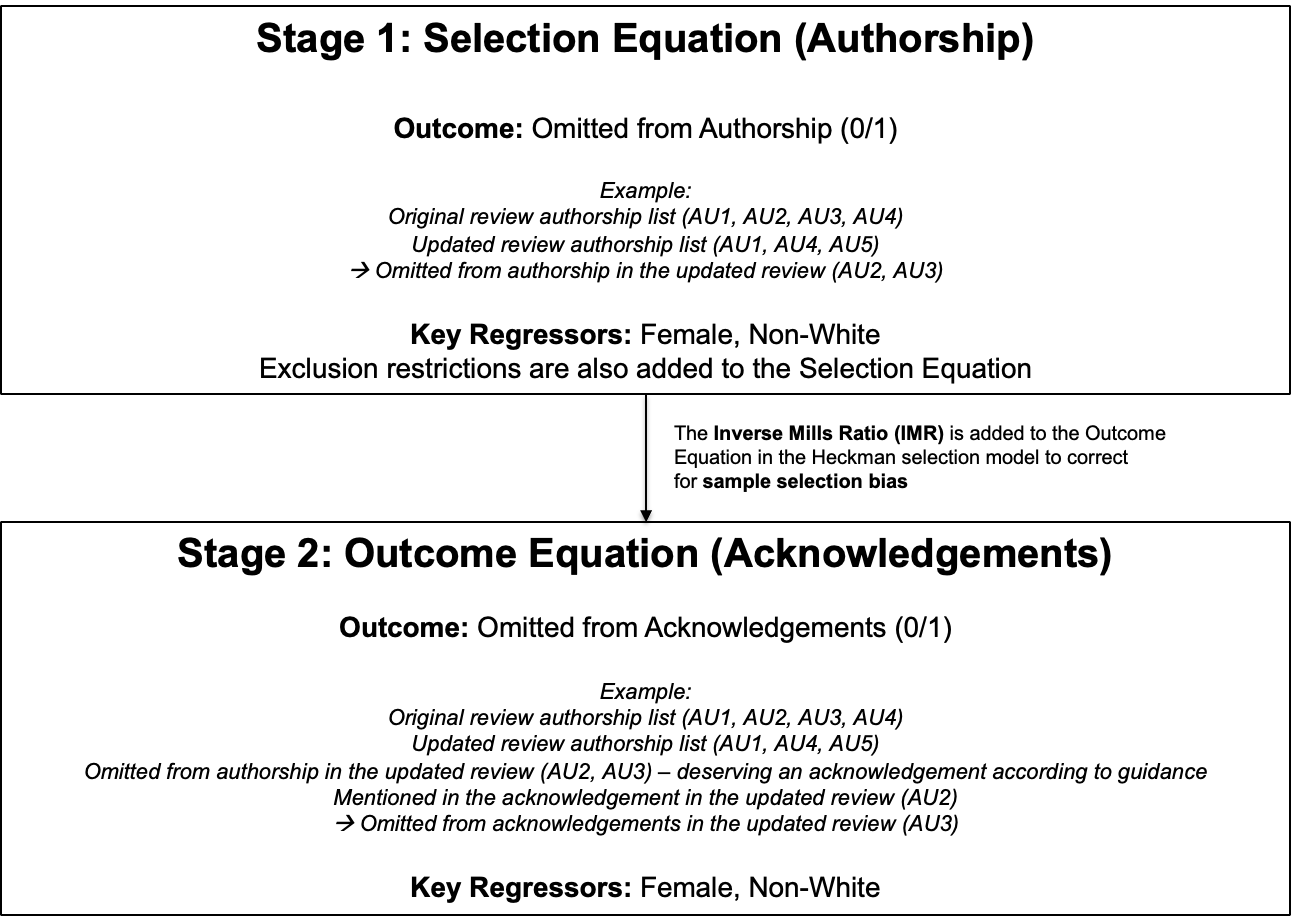
**

**Fig S1C. Methodological framework for the Two-Stage analysis.**

The figure outlines the sequential logic of the analysis: Stage 1 models the probability of omission from authorship; Stage 2 models the probability of omission from acknowledgements, including the Inverse Mills Ratio from Stage 1 to correct for selection bias.
