# Supplementary material for: Attribution of credit in acknowledgements: The case of systematic reviews in medicine
Source: PLoS One. 2026 Jan 6;21(1):e0338714. doi: 10.1371/journal.pone.0338714 (PMC12773806; doi:10.1371/journal.pone.0338714)
Supplement: S2 File — (DOCX) [file pone.0338714.s002.docx]

**S2 File. Supplemental tables.**

**Table S2A. Correlates of Omission from the acknowledgements – interactions.**

|  | **Omission from**  **authorship** | | **Omission from**  **acknowledgements** | |
| --- | --- | --- | --- | --- |
|  | **(1)** | **(2)** | **(3)** | **(4)** |
| **Variables** | **Intersection.** | **Contribution** | **Intersection.** | **Contribution** |
| Female x Non-White | 0.95 |  | 1.13 |  |
|  | (0.07) |  | (0.10) |  |
| Male x Non-White | 0.96 |  | 1.24*** |  |
|  | (0.06) |  | (0.09) |  |
| Female x White | 0.96 |  | 1.05 |  |
|  | (0.04) |  | (0.06) |  |
| Female |  | 0.94 |  | 1.01 |
|  |  | (0.04) |  | (0.05) |
| Minor Contribution x Non-White |  | 1.30** |  | 1.06 |
|  |  | (0.13) |  | (0.13) |
| Major Contribution x Non-White |  | 0.95 |  | 1.15* |
|  |  | (0.05) |  | (0.08) |
| Minor Contribution x White |  | 1.18*** |  | 1.04 |
|  |  | (0.07) |  | (0.08) |
|  |  |  |  |  |
| Observations | 7,752 | 6,793 | 7,752 | 6,793 |

Notes: Std. Err. adjusted for clusters by author ID and review ID. Cochrane group dummies and year dummies included. All controls are included and not reported for parsimony. Columns 2 and 4 have fewer observations because we could only attach a minor or a major contribution to 6,793 observations. Odds ratio reported. *** p<0.01, ** p<0.05, * p<0.1.

**Table S2B. Keywords used to categorise review contributions.**

| **Minor/technical contributions** | **Major/conceptual contributions** |
| --- | --- |
| "extract", "retrieve", "abstract", "screen", "provide", "collect", "enter", "obtain", "acquire", "search", "manage", "input", "extracting", "retrieving", "abstracting", "screening", "providing", "collecting", "entering", "obtaining", "assessment" "acquiring", "searching", "managing", "inputing", "extracted", "retrieved", "abstracted", "screened", "provided", "collected", "entered", "obtained", "acquired", "searched", "managed", "inputed", "data", "literature", "paper", "study", "studies", | "draft", "write", "advise", "revise", "conceive", "analyse", "analyze", "analysis", "comment", "design", "guarantor", "advisor", "assessor", "contact", "finance", "interpret", "coordinate", "drafting", "writing", "advising", "revising", "conceiving", "analysing", "analysing", "reviewing", "commenting", "designing", "guarantor", "advising", "assessing", "contacting", "financing", "interpreting", "coordinating", "drafted", "wrote", "advised", "revised", "conceived", "analysed", "analyzed", "reviewed", "commented", "designed", "guarantor", "advised", "assessed", "contacted", "financed", "interpreted", "coordinated", "concept", "manuscript", "version", "interpretation", "protocol", "updating", "updated", "advice", "perspective", "report", "edited", "editing" |

**Table S2C. Examples of acknowledgement texts.**

| **Type of acknowledgements** | **Example** |
| --- | --- |
| Previous review authors acknowledged but not individually named | "We would like to thank the authors of included trials who provided individual patient data. We would also like to thank the authors of the previous version of this review." |
|  | "We would like to thank the authors of previous editions of this review and we are very grateful for their hard work and enthusiasm." |
| Other individuals acknowledged | "We would like to thank […] for her assistance with the searches for this review. We would also like to thank […] and […] for their helpful comments." |
|  | "The authors thank […] for providing unpublished trial data." |
| Consumer reviewers acknowledged | "We are grateful to the Editorial Board of the Cochrane Stroke Group, external peer reviewer, and consumer reviewer […] for making constructive comments on this review. No pharmaceutical company was involved in this review." |
|  | "The review authors would like to acknowledge the Cochrane Dementia and Cognitive Improvement Group for their help as well as […] for her contribution as consumer editor." |
| Translators acknowledged | "Many thanks to […] for providing a translation for Sakamoto 1985." |
|  | "Thank you to the primary authors who responded to requests for information. We also thank our volunteers, our translators and the Cochrane Back Review Group editors." |
| Funding acknowledged | “Funding for the IBD/FBD Review Group has been provided by the Canadian Institutes of Health Research (CIHR) Knowledge Translation Branch.” |
|  | “The review authors wish to thank the Common Mental Disorder Group Information Specialist for assistance in developing the search strategy. CRG Funding Acknowledgement The National Institute for Health Research (NIHR) is the largest single funder of the Cochrane Common Mental Disorders Group.” |

**Further analysis**.

We ran additional tests that considered team-level characteristics (see **Table S2D** below). First, we examined race differences within the team based on the race of all authors in the original review. High values of this score indicate that the contributor’s race differed substantially from that of other team members. The coefficient on Difference in race x Non-White (OR=0.60 in column 4) indicates that non-White contributors were less likely to be missing from the acknowledgements in White-dominated teams

Second, we explored homophily between the focal scientist and the team’s lead. As a common practice in medicine, we considered the corresponding author of the updated review to be the research lead. First and/or last authors may also oversee the writing of the manuscript in the life sciences. In our sample, in 92% of the cases, the corresponding author was the first (75%) or last (17%) author. Informal conversations with Cochrane review authors also confirmed that the corresponding author typically oversees the review. Contrary to expectations of homophily effects, we found that Non-White collaborators were more likely to be missing from acknowledgements when the corresponding author was non-White (OR = 1.26 in column 5).

Finally, we considered the location of the corresponding author of the updated review to assign it to “North America, Europe and Oceania” or “Asia, South America, and Africa”. We found that relative to White authors in reviews from North America, Europe, and Oceania, non-White authors in reviews from Asia, South America, and Africa were more likely to be excluded (OR=1.27 in column 6).

**Table S2D. Correlates of Omission from the acknowledgements – interactions.**

|  | **Omission from**  **authorship** | | |  | **Omission from acknowledgements** |  |
| --- | --- | --- | --- | --- | --- | --- |
|  | **(1)** | **(2)** | **(3)** | **(4)** | **(5)** | **(6)** |
| **Variables** | **Difference in race** | **Homophily** | **Geography** | **Difference in race** | **Homophily** | **Geography** |
|  | 0.97 | 0.97 | 0.97 | 1.02 | 1.02 | 1.01 |
| Female | (0.04) | (0.04) | (0.04) | (0.05) | (0.05) | (0.05) |
|  |  |  |  |  |  |  |
| Non-White | 0.94 |  |  | 1.54*** |  |  |
|  | (0.08) |  |  | (0.17) |  |  |
| Difference in race | 1.11 |  |  | 1.08 |  |  |
|  | (0.08) |  |  | (0.11) |  |  |
| Difference in race x Non-White | 1.00 |  |  | 0.60*** |  |  |
|  | (0.15) |  |  | (0.11) |  |  |
| Non-White lead x Non-White |  | 0.87** |  |  | 1.26*** |  |
|  |  | (0.06) |  |  | (0.10) |  |
| White lead x Non-White |  | 1.20*** |  |  | 1.06 |  |
|  |  | (0.07) |  |  | (0.08) |  |
| Non-White lead x White |  | 1.20*** |  |  | 0.94 |  |
|  |  | (0.06) |  |  | (0.07) |  |
| Asia, South America and Africa (UR) x Non-White |  |  | 0.97 |  |  | 1.27*** |
|  |  |  | (0.06) |  |  | (0.10) |
| North America, Europe and Oceania (UR) x Non-White |  |  | 1.04 |  |  | 1.09 |
|  |  |  | (0.06) |  |  | (0.08) |
| Asia, South America and Africa (UR) x White |  |  | 1.18** |  |  | 0.91 |
|  |  |  | (0.08) |  |  | (0.09) |
|  |  |  |  |  |  |  |
| Observations | 7,752 | 7,752 | 7,752 | 7,752 | 7,752 | 7,752 |

Notes: Std. Err. adjusted for clusters by author ID and review ID. Cochrane group dummies and year dummies included. All controls are included and not reported for parsimony. Odds ratio reported. *** p<0.01, ** p<0.05, * p<0.1.

We also extracted the institutional affiliations of the focal individuals and matched them to either “Asia, South America, and Africa” or “North America, Europe, and Oceania”. We were able to attach this information to 7,559 of 7,752 individuals. Out of these, 1,219 (16.1%) were from Asia, South America, or Africa. Interestingly, this is only moderately correlated with our Non-White variable (r = 0.51). To unpack the effects of race and geography, we included this variable in our model (see **Table S2E** below). The coefficient on Non-White remains substantially unchanged. The coefficient on the “Asia, South America and Africa affiliation” is not statistically significant in predicting Omission from acknowledgements (OR=1.06 in column 2). This suggests that the effect of race trumps that of geography in shaping omission from the acknowledgements. Given the high correlations between “Asia, South America and Africa affiliation” and “Asia, South America and Africa (UR)”, the results below should be interpreted with caution.

**Table S2E. Correlates of Omission from the acknowledgements.**

|  | **With additional control:**  **Asia, South America and Africa affiliation** | |
| --- | --- | --- |
|  | **(1)** | **(2)** |
|  | **Omission from**  **authorship** | **Omission from**  **acknowledgements** |
| Female | 0.96 | 1.02 |
|  | (0.04) | (0.05) |
| Non-White | 1.01 | 1.15** |
|  | (0.05) | (0.07) |
| Academic influence | 0.95*** | 1.01 |
|  | (0.01) | (0.02) |
| Asia, South America and Africa affiliation | 0.83** | 1.06 |
|  | (0.07) | (0.11) |
| First author | 0.44*** | 0.91 |
|  | (0.02) | (0.08) |
| Last author | 0.79*** | 0.94 |
|  | (0.03) | (0.05) |
| Institutional status | 1.00 | 1.00 |
|  | (0.00) | (0.00) |
| Research protocol | 0.68** | 1.09 |
|  | (0.11) | (0.22) |
| Institutional status team lead | 1.00*** | 1.00** |
|  | (0.00) | (0.00) |
| External funding | 1.10** | 1.05 |
|  | (0.05) | (0.06) |
| Length acknowledgements | 1.15*** | 0.74*** |
|  | (0.03) | (0.02) |
| Authors count | 0.92*** | 1.07*** |
|  | (0.01) | (0.01) |
| Asia, South America and Africa (UR) | 1.18** | 0.99 |
|  | (0.08) | (0.09) |
| Years difference | 1.09*** |  |
|  | (0.01) |  |
| No Recent publication | 2.17*** |  |
|  | (0.11) |  |
| Inverse Mills Ratio |  | 0.36*** |
|  |  | (0.03) |
|  |  |  |
| Observations | 7,559 | 7,559 |

Notes: Std. Err. adjusted for clusters by author ID and review ID. Cochrane group dummies and year dummies included. Odds ratio reported. *** p<0.01, ** p<0.05, * p<0.1.

**Distribution of contributors omitted from the acknowledgements.**

In the main manuscript, we report that 88 % of omissions occurred in reviews that failed to mention any prior contributors, while only 12 % involved selective exclusion. We attempted to re-estimate the model after recoding “Omission from the acknowledgements” as 1 only for the 72 individuals who were selectively omitted, and 0 otherwise. However, because these treated cases represent less than 1% of the full sample, several control variables perfectly predicted the outcome, leading to a substantial loss of observations (approximately 40%) in the second stage. Given the instability of the estimates under this operationalisation, we instead conducted two-sample tests of proportions to compare Female and Non-White contributors across wholesale and discerning omission cases. As reported in **Table S2F** below, there is no significant difference in the proportion of females. Non-White individuals, however, are more represented in the “wholesale omissions” group, which refers to cases where no prior contributors were acknowledged at all.

**Table S2F. Two-sample test of proportions.**

|  | **Wholesale omissions**  **n=549** | **Discerning omissions**  **n=72** | **Difference** | **Pr (Z < z)** |
| --- | --- | --- | --- | --- |
| Female | 0.45 | 0.53 | -0.07 | 0.12 |
| Non-White | 0.26 | 0.15 | 0.11 | 0.02 |

**Table S2G. Robustness checks.**

|  | **Removing**  **citation**  **outliers** | | **Removing**  **review**  **protocols** | | **Removing individual with probability of accurate**  **gender < 50%** | | **Adding “Productivity” and “Cochrane experience”**  **controls** | | **Adding**  **“Acknowledges others besides prior review authors”**  **control** | | **Entering individual**  **race**  **subgroups** | |
| --- | --- | --- | --- | --- | --- | --- | --- | --- | --- | --- | --- | --- |
|  | **(1)** | **(2)** | **(3)** | **(4)** | **(5)** | **(6)** | **(7)** | **(8)** | **(9)** | **(10)** | **(11)** | **(12)** |
| **Variables** | **Omission from authorship** | **Omission from acknowledgements** | **Omission from authorship** | **Omission from acknowledgements** | **Omission from authorship** | **Omission from acknowledgements** | **Omission from authorship** | **Omission from acknowledgements** | **Omission from authorship** | **Omission from acknowledgements** | **Omission from authorship** | **Omission from acknowledgements** |
|  |  |  |  |  |  |  |  |  |  |  |  |  |
| Female | 0.98 | 1.03 | 0.96 | 1.02 | 0.95 | 0.97 | 0.96 | 1.01 | 0.97 | 1.01 | 0.97 | 1.01 |
|  | (0.04) | (0.05) | (0.04) | (0.05) | (0.04) | (0.05) | (0.04) | (0.05) | (0.04) | (0.05) | (0.04) | (0.05) |
| Non-White | 0.99 | 1.17** | 0.97 | 1.17** | 0.95 | 1.12* | 1.00 | 1.18*** | 0.97 | 1.17*** |  |  |
|  | (0.05) | (0.07) | (0.05) | (0.07) | (0.05) | (0.08) | (0.05) | (0.07) | (0.05) | (0.07) |  |  |
| Publication exper. |  |  |  |  |  |  | 0.97 | 1.02 |  |  |  |  |
|  |  |  |  |  |  |  | (0.02) | (0.03) |  |  |  |  |
| Cochrane exper. |  |  |  |  |  |  | 0.96 | 1.01 |  |  |  |  |
|  |  |  |  |  |  |  | (0.04) | (0.05) |  |  |  |  |
| Acknowledges oth. |  |  |  |  |  |  |  |  | 1.06 | 0.99 |  |  |
|  |  |  |  |  |  |  |  |  | (0.07) | (0.08) |  |  |
| Asian |  |  |  |  |  |  |  |  |  |  | 0.98 | 1.15** |
|  |  |  |  |  |  |  |  |  |  |  | (0.05) | (0.08) |
| Hispanic |  |  |  |  |  |  |  |  |  |  | 1.08 | 1.27** |
|  |  |  |  |  |  |  |  |  |  |  | (0.09) | (0.12) |
| Black |  |  |  |  |  |  |  |  |  |  | 0.66*** | 1.00 |
|  |  |  |  |  |  |  |  |  |  |  | (0.10) | (0.24) |
| Observations | 6,993 | 6,993 | 7,427 | 7,427 | 6,317 | 6,317 | 7,752 | 7,752 | 7,752 | 7,752 | 7,752 | 7,752 |

Notes: Std. Err. adjusted for clusters by author ID and review ID. All controls are included and not reported for parsimony. Cochrane group dummies and year dummies included. Odds ratio reported. *** p<0.01, ** p<0.05, * p<0.1.
