# Supplementary material for: Attribution of credit in acknowledgements: The case of systematic reviews in medicine
Source: PLoS One. 2026 Jan 6;21(1):e0338714. doi: 10.1371/journal.pone.0338714 (PMC12773806; doi:10.1371/journal.pone.0338714)
Supplement: Attachment — Submitted filename: Response_to_reviewers.docx [file pone.0338714.s004.docx]

**Response to Reviewers for “Attribution of credit in acknowledgements: The case of systematic reviews in medicine” [PONE-D-25-29842].**

**Editor’s comments:**

Opening comment: Your manuscript was sent to three reviewers with strong and

complementary expertise. I also read the manuscript independently and I agree with their

assessment. This is a well-conceived and well-written paper, however, there is room to

strengthen the manuscript on several fronts.

**Response:** We are delighted that your assessment of the paper, in agreement with the reviewers’, is favourable. We appreciate the opportunity to submit a revised version.

1. On the empirical side, each reviewer highlights very actionable aspects to make the results more robust. Please address these points carefully.

**Response:** We have taken all reviewers’ comments concerning the empirics to heart. In particular, following Reviewer 1’s advice, we assessed the robustness of our findings to the inclusion of new control variables and improved our analysis of the acknowledgement texts by, for example, considering whether a review acknowledged others besides the prior review authors. As recommended by Reviewer 2, we elaborated on the gender and race classification algorithms and improved the operationalisation of the ‘Institutional status’ variable. As suggested by Reviewer 3, we also created two additional figures that summarise the study methods and the process of constructing our dataset.

2. Regarding the conceptual framework, the paper would benefit from a more developed background discussion on the role and use of acknowledgements in scholarly communication. In its current form, the “Related Work” section touches on central aspects but does not fully elaborate them. Expanding this section by engaging with themes such as the implicit codes of professional and normative behaviour acknowledgements follow, the variation in co- and sub-authorship practices across research domains, and the under-recognised role of acknowledgements within the reward system of science, would significantly strengthen the positioning and contribution of the paper. I provide some references below that may be helpful in this regard.

Baccini, A., & Petrovich, E. (2021). Normative versus strategic accounts of acknowledgment data: The case of the top-five journals of economics. Scientometrics.[https://doi.org/10.1007/s11192-021-04185-6](https://eur01.safelinks.protection.outlook.com/?url=https%3A%2F%2Fdoi.org%2F10.1007%2Fs11192-021-04185-6&data=05%7C02%7Crs2406%40bath.ac.uk%7C1a9d59720b454e2ee38b08ddfa76ed98%7C377e3d224ea1422db0ad8fcc89406b9e%7C0%7C0%7C638942111182230916%7CUnknown%7CTWFpbGZsb3d8eyJFbXB0eU1hcGkiOnRydWUsIlYiOiIwLjAuMDAwMCIsIlAiOiJXaW4zMiIsIkFOIjoiTWFpbCIsIldUIjoyfQ%3D%3D%7C0%7C%7C%7C&sdata=PkJ2UGjURG7ktsTqSj7lC8gUGMqTrF%2FGqkLP25W2g4w%3D&reserved=0)

Cronin, B. (1995), The Scholar’s Courtesy: The Role of Acknowledgments in the Primary Communication Process, Taylor Graham, Los Angeles, CA.

Díaz-Faes, A. A., & Bordons, M. (2017). Making visible the invisible through the analysis of acknowledgements in the humanities. Aslib Journal of Information Management, 69(5), 576–590.[https://doi.org/10.1108/AJIM-01-2017-0008](https://eur01.safelinks.protection.outlook.com/?url=https%3A%2F%2Fdoi.org%2F10.1108%2FAJIM-01-2017-0008&data=05%7C02%7Crs2406%40bath.ac.uk%7C1a9d59720b454e2ee38b08ddfa76ed98%7C377e3d224ea1422db0ad8fcc89406b9e%7C0%7C0%7C638942111182242536%7CUnknown%7CTWFpbGZsb3d8eyJFbXB0eU1hcGkiOnRydWUsIlYiOiIwLjAuMDAwMCIsIlAiOiJXaW4zMiIsIkFOIjoiTWFpbCIsIldUIjoyfQ%3D%3D%7C0%7C%7C%7C&sdata=nP6Fwtt96L%2FD%2Fk6gPMEigkFOeL0cBBbRWsb%2F0FJsp%2Bo%3D&reserved=0)

Hyland, K. (2003), “Dissertation acknowledgments: the anatomy of a Cinderella genre”, Written Communication, Vol. 20 No. 3, pp. 242-268.

Mccain, K. W. (1991). Communication, Competition, The Production and Secrecy : and Dissemination of Research-Related in Genetics Information. Science, Technology, & Human Values, 16(4), 491–516.

**Response:** Thank you for encouraging us to refine the conceptual framework and for directing us to these insightful papers. We read them with interest and incorporated them into the manuscript.

We have expanded the “Related Work” section to discuss how, unlike more institutionalised mechanisms within the scientific reward system, such as authorship and citations, acknowledgements follow largely uncodified norms. We also highlight the nuanced nature of acknowledgement texts, which offer a unique window into research practices and interactions that may not be evident by simply examining authorship bylines (e.g., McCain, 1991).

In the “Attribution of credit for non-author contributors via acknowledgements” subsection, we engage with research that shows disciplinary and geographical variation in the prevalence of acknowledgements and their forms (e.g., Díaz-Faes & Bordons, 2014). We also created a specific subsection titled “The under-recognised role of acknowledgements”, which highlights reasons why acknowledgements have received little attention, and why this ‘Cinderella genre’ (Hyland, 2003) is instead worthy of consideration.

Moreover, following Reviewer 3’s advice, we highlighted a subsection titled “The Impact of Gender and Race on Acknowledgement Practices” to set the scene for the paper’s key question about inequalities.

We believe that, as a result of these changes, the paper’s conceptual framework is stronger and that these revisions have led to a more effective positioning and contribution to the study.

As Reviewer 2 asked us to streamline the literature review to reduce overlap with the introduction, we have tried to keep these changes concise but remain ready to make more modifications if deemed helpful.

Many thanks for your guidance.

**Reviewers' comments:**

**Reviewer #1:**

Opening Comment. The paper makes an important contribution to the study of inequalities in science, supplementing prior work on recognition in authorships and citation by gender, race, region, etc. by looking at acknowledgements. Cochrane reviews provide a good setting for looking for demographic differences in acknowledgment.

**Response:** We are pleased that you thought the paper’s contribution is important and that you shared our interest in exploring Cochrane as a suitable setting.

1. You might also want to discuss more why seniority/position does not affect acknowledgement, even though it effects authorship (cf. Lissoni, et al., 2013, which you cite).

**Response:** We fully agree on this point, as this finding was also puzzling for us.

Although not statistically significant at conventional levels, the odds ratios for First Author and Last Author are lower than 1 in the second stage, suggesting that, directionally, authors occupying these positions might be less likely to be omitted relative to the baseline category, Middle Author. To provide further discussion for these non-significant findings, we have enhanced the manuscript text by incorporating two possible interpretations.

First, we suggest that power dynamics and hierarchical norms, which typically shape authorship decisions, may be less salient in the case of acknowledgements. There may be fewer incentives to include senior individuals out of deference or obligation, as acknowledgements are less closely tied to career outcomes or prestige than authorship. Second, we argue that the rules of attribution for acknowledgements are looser than those for authorship, which may give the author greater autonomy and reduce the influence of seniority or institutional politics.

2. Controlling for average cites is good. How about controlling for total pubs (or total in last three years) as another measure of visibility?

**Response:** Many thanks for this suggestion. We agree that considering the publication count as a measure of the focal contributor's productivity is helpful. To capture this, we created the variable “Productivity”, which corresponds to the log transformation of the average yearly publication of the focal individual (total publications divided by academic age). We use yearly counts, rather than total counts, to separate the effect of academic age. For example, an individual might have more publications simply because they have been in academia for a longer period.

We also thought it might be helpful to consider Cochrane publications alone, as a measure of visibility and experience within the Cochrane network. We constructed the variable “Cochrane experience”, which corresponds to the log transformation of the cumulative number of Cochrane publications for the focal individual.

As expected, these variables are positively correlated with our measure of academic influence (0.64 and 0.27, respectively) and with each other (0.48):

**Descriptive statistics.**

|  |  | **Mean** | **St. Dev.** | **1** | **2** |
| --- | --- | --- | --- | --- | --- |
| 1 | Academic influence (Yearly citations) | 42.37 | 87.96 |  |  |
| 2 | Productivity (Yearly publications) | 4.30 | 4.42 | 0.64 |  |
| 3 | Cochrane experience (Cochrane publications) | 13.40 | 21.91 | 0.27 | 0.48 |

To verify whether our results would vary with the inclusion of these controls, we entered both variables (“Productivity” and “Cochrane experience”) into our model and obtained the results shown in columns 3 and 4 below. The key finding that non-White contributors are significantly more likely to be omitted than their White counterparts is largely unchanged in terms of significance and direction:

**Correlates of omission from the acknowledgements.**

|  | **Original model** | | **With additional controls: Productivity and**  **Cochrane experience** | |
| --- | --- | --- | --- | --- |
|  | **(1)** | **(2)** | **(3)** | **(4)** |
|  | **Omission from**  **authorship** | **Omission from acknowledgements** | **Omission from**  **authorship** | **Omission from acknowledgements** |
|  |  |  |  |  |
| Female | 0.97 | 1.01 | 0.96 | 1.01 |
|  | (0.04) | (0.05) | (0.04) | (0.05) |
| Non-White | 0.97 | 1.17*** | 1.00 | 1.18*** |
|  | (0.05) | (0.07) | (0.05) | (0.07) |
| Academic influence | 0.95*** | 1.01 | 0.82*** | 1.00 |
|  | (0.01) | (0.02) | (0.04) | (0.06) |
| **Productivity** |  |  | **0.97** | **1.02** |
|  |  |  | **(0.02)** | **(0.03)** |
| **Cochrane experience** |  |  | **0.96** | **1.01** |
|  |  |  | **(0.04)** | **(0.05)** |
|  |  |  |  |  |
| Observations | 7,752 | 7,752 | 7,752 | 7,752 |

Note: All other control variables were included and not reported here.

Given the high correlation values, we decided to retain “Academic influence” alone in the main model; however, we have now added these controls to the robustness checks reported in Table G of S2 File. In the manuscript text, we note that these additional analyses confirm that our key finding remains robust even with the inclusion of productivity and experience controls.

3. How robust are your result to recoding the generic acknowledgements (“We acknowledge the work of the author team for the first version of the review”.) as 0 instead of 1?

**Response:** We should clarify that these generic acknowledgement cases are currently being treated as ‘proper’ acknowledgements and are accordingly already coded as 0 (where 1 indicates omission). This is a conservative approach because it assumes that no one was omitted if the updated review acknowledged all previous authors (even if they are not individually named).

We should also note that these cases are sporadic, accounting for only 25 of 7,752 observations (0.32% of the sample). Nevertheless, as a robustness check, we removed the 25 cases from the sample. Our main results remain unchanged, as reported here:

**Correlates of omission from the acknowledgements.**

|  | **Full sample** | | **Without “Generic acknowledgements”** | |
| --- | --- | --- | --- | --- |
|  | **(1)** | **(2)** | **(3)** | **(4)** |
|  | **Omission from**  **authorship** | **Omission from acknowledgements** | **Omission from**  **authorship** | **Omission from acknowledgements** |
|  |  |  |  |  |
| Female | 0.97 | 1.01 | 0.97 | 1.01 |
|  | (0.04) | (0.05) | (0.04) | (0.05) |
| Non-White | 0.97 | 1.17*** | 0.96 | 1.18*** |
|  | (0.05) | (0.07) | (0.05) | (0.07) |
|  |  |  |  |  |
| Observations | 7,752 | 7,752 | 7,727 | 7,727 |

Note: All other control variables were included and not reported here.

Our original text might have caused confusion, so we have amended it as follows: ‘Finally, we determined whether the acknowledgements contained sentences such as “We acknowledge the work of the author team for the first version of the review”. Even if the individuals were not named, we treat these cases (n=25 out of 7,752 observations, or 0.32% of the sample) as a proper acknowledgement (Omission from acknowledgements = 0).’

4. What happens if you add a new variable, “Acknowledges others besides prior review authors”? And see what review team variables predict this (same as what predicts dropping prior authors, but with sign reversed)? And, if you then control for this variable, does it affect your current results? This can be seen as a measure of how generous/stingy the author of the new review is, and might explain some of the drops in acknowledgements (beyond the demographics of prior authors).

**Response:** Thank you for this suggestion. To create this new variable, we counted the total number of names mentioned in each acknowledgements text and removed from this count the number of prior authors mentioned in the same text. We then created a dummy variable, “Acknowledges others besides prior review authors”, equal to 1 if the number of remaining acknowledged names was greater than zero, and 0 otherwise. For 7,019 out of the 7,752 observations (90.5%), this variable equals 1, suggesting that most reviews do indeed acknowledge others besides prior review authors.

As our main dependent variable, “Omitted from the acknowledgements”, is at the individual level (whether or not the focal individual is omitted), and this new variable is at the review level (whether or not the focal review acknowledges others), they are not directly comparable. However, to answer your question of what variables might predict whether a review acknowledges others besides prior review authors, we checked correlations with other review-level variables. As reported in the table below, no correlation value stands out, except, as expected, the positive correlation with the length of the acknowledgements.

**Correlations.**

|  |  | **1** | **2** | **3** | **4** | **5** | **6** |
| --- | --- | --- | --- | --- | --- | --- | --- |
| 1 | Acknowledges others besides prior authors |  |  |  |  |  |  |
| 2 | Research protocol | -0.03 |  |  |  |  |  |
| 3 | Institutional status team lead | 0.03 | -0.07 |  |  |  |  |
| 4 | External funding | 0.08 | -0.12 | 0.10 |  |  |  |
| 5 | Length acknowledgements | 0.45 | -0.12 | 0.08 | 0.13 |  |  |
| 6 | Authors count | 0.03 | -0.07 | 0.06 | 0.03 | 0.12 |  |
| 7 | Asia, South America and Africa | -0.02 | -0.02 | -0.26 | -0.01 | -0.01 | -0.07 |

Following your advice, we also checked whether controlling for this variable would affect our main results. As reported in the table below, our key finding (that non-White contributors are significantly more likely to be omitted) is robust to the inclusion of this control. We have added this to our battery of robustness checks reported in Table G in the S2 File.

**Correlates of omission from the acknowledgements.**

|  | **Original model** | | **With additional control:**  **Acknowledges others besides prior review authors** | |
| --- | --- | --- | --- | --- |
|  | **(1)** | **(2)** | **(3)** | **(4)** |
| **VARIABLES** | **Omission from**  **authorship** | **Omission from acknowledgements** | **Omission from**  **authorship** | **Omission from acknowledgements** |
|  |  |  |  |  |
| Female | 0.97 | 1.01 | 0.97 | 1.01 |
|  | (0.04) | (0.05) | (0.04) | (0.05) |
| Non-White | 0.97 | 1.17*** | 0.97 | 1.17*** |
|  | (0.05) | (0.07) | (0.05) | (0.07) |
| Acknowledges others |  |  | 1.06 | 0.99 |
|  |  |  | (0.07) | (0.08) |
| Observations | 7,752 | 7,752 | 7,752 | 7,752 |

Note: All other control variables were included and not reported here.

5. For the effect of length, you might want to exclude the author names from the length count, as these are mechanically correlated with the outcome (in the wrong direction).

**Response:** Thank you for this observation. We agree that the length of the acknowledgement texts might be negatively correlated with our key outcome, the omission of the focal individual. If someone is omitted, the length of the acknowledgements will be naturally lower because their name is not included within it.

To mitigate this issue, we followed your advice and removed all names from the acknowledgement text before calculating the length. As expected, the length of this non-person-related acknowledgement text is highly correlated with our original variable (0.92).

We now use this new operationalisation of “Length of acknowledgements” in all our models and recalculated all coefficients. Our key results are largely unaffected.

6. “Following Certo et al. [43], to assess the strength of our exclusion restrictions, we checked) the correlation between the key independent variables of interest in the second stage (Female and Non-White) and the Inverse Mills Ratio, as well as the pseudo-R2 associated with the first stage.“ What do the correlations show?

**Response:** We appreciate your request for clarification. We report all relevant correlation coefficients and pseudo-R2 values in the table below.

**Correlations with Inverse Mills Ratio (IMR).**

|  | **No**  **exclusion restrictions** | **With “Years difference” and “No recent publication” as exclusion restrictions** |
| --- | --- | --- |
| Corr [x_1_, IMR]  x_1_= Female | 0.047 | 0.031 |
| Corr [x_2_, IMR]  x_2_= Non-White | 0.045 | 0.046 |
| pseudo-R² | 0.11 | 0.16 |

Note. The correlations between x_1_, x_2_ and the Inverse Mills Ratio are stated in absolute terms.

When no exclusion restrictions are added to the model, Corr [Female, IMR] = 0.047, Corr [Non-White, IMR] = 0.045 and the pseudo-R² = 0.11. When we add the exclusion restrictions, Corr [Female, IMR] decreases to 0.031, Corr [Non-White, IMR] decreases to 0.046, and the pseudo-R² increases to 0.16. These measures illustrate the effectiveness of the chosen exclusion restrictions.

We have added this interpretation to the Results section: “Following Certo et al. (2016), we examine the correlation between our key regressors and the Inverse Mills Ratio as an indicator of exclusion restrictions’ strength. The low correlations (ranging from 0.031 to 0.047) suggest that the chosen exclusion restrictions are effective. At the same time, the increase in the pseudo-R² of the first-stage model when including the exclusion restrictions (from 0.11 to 0.16) indicates that the exclusion restrictions enhance the model fit.”

7. What happens when you control for the continent of the Original Review? You report the ContinentxRace interactions in the Further Analyses. But, what is the main effect of the original review being from Asia-South America-Africa? Put differently, are the URs discriminating on the basis of race or geography?

**Response:** We appreciate this valuable suggestion. We have clarified in the text that the current “Asia, South America and Africa” variable refers to the corresponding author of the updated review (UR), not the original review (OR). This might have been clear in the table, where we use the “UR” annotation, but could have been made more explicit in the text, so we have addressed this issue.

Although we could control for the continent of origin in the original review team, we feel that this would not address the question of whether the updated review teams discriminate based on geography. Instead, to test whether omission from acknowledgements differs by geography, we extracted the institutional affiliations of each focal contributor from the original review and classified them by continent (“Asia, South America, and Africa” vs. “North America, Europe, and Oceania”).

We matched this information for 7,559 of 7,752 individuals (97.5%). Out of these, only 1,219 authors (16.1%) were affiliated with institutions in Asia, South America, or Africa. Interestingly, the correlation between this variable and the “Non-White” indicator was moderate (correlation=0.51), suggesting that non-White individuals in our sample are not necessarily affiliated with institutions operating from “Asia, South America, or Africa”. This might be the case, for example, for a colleague with a “Non-White” name (as classified by the name-based tool) working in the UK.

In the table below, we present a small set of observations from our sample to illustrate different combinations of race (Non-White = 0, 1) and geography (Asia, South America, or Africa = 0, 1).

**Example observations with different combinations of Non-White and Asia, South America, or Africa.**

| **Combination** | **Original Review Author Name** | **Original Review Author Affiliation** | **Non-White** | **Asia, South America or Africa** |
| --- | --- | --- | --- | --- |
| **0, 0** | Clive E Adams | Cochrane Schizophrenia Group, University of Nottingham, Nottingham, UK | 0 | 0 |
|  | Anthony David | Institute of Psychiatry, BOX PO68, London, UK | 0 | 0 |
|  | Vanessa J Poustie | Institute of Child Health, University of Liverpool, Royal Liverpool Children's Hospital NHS Trust, Liverpool, UK | 0 | 0 |
| **0, 1** | Airton T Stein | Department of Epidemiology, Fundacao Faculdade Federal de Ciencias Medicas de Porto Algre, Brazil | 0 | 1 |
|  | Mayer Brezis | Department of Medicine, Hadassah Hebrew University Hospital, Jerusalem, Israel | 0 | 1 |
|  | Durrane Z Thaver | Department of Paediatrics, The Aga Khan University Hospital, Karachi, Pakistan | 0 | 1 |
| **1, 0** | Sachiendra V Amaragiri | Northern Vascular Centre, Freeman Hospital, Newcastle upon Tyne, UK | 1 | 0 |
|  | Wei Shen Lim | Department of Respiratory Medicine, Nottingham University Hospitals Trust, Nottingham, UK | 1 | 0 |
|  | Samir K Ballas | Department of Medicine, Division of Hematology, Thomas Jefferson University, Philadelphia, USA. | 1 | 0 |
| **1, 1** | Yoshihiro Kinoshita | Department of Psychiatry & Cognitive_Behavioral Medicine, Nagoya City University Graduate School of Medical Sciences, Nagoya, Japan | 1 | 1 |
|  | Rungsan Chaisewikul | Department of Medicine, Mahidol University, Bangkok, Thailand | 1 | 1 |
|  | Kelvin H Tan | Department of Maternal Fetal Medicine, KK Women's and Children's Hospital, Singapore | 1 | 1 |

To unpack the effects of race and geography, we conducted robustness checks by including the “Asia, South America, or Africa affiliation” variable in our model. We checked the corresponding coefficients when the variable is entered in the model and “Non-White” is included (see columns 3-4 in the table below) or excluded (see columns 5-6). The coefficient on “Non-White” remains substantially unchanged, and the coefficient on the “Asia, South America and Africa affiliation” is not statistically significant in predicting the second stage “Omission from acknowledgements”. This suggests that the effect of race trumps that of geography in shaping omission from the acknowledgements.

**Main results with additional control.**

|  | **Original model** | | **With additional control:**  **Asia, SA and Africa affiliation** | | **With additional control:**  **Asia, SA and Africa affiliation but removing Non-White** | | **With additional control:**  **Asia, SA and Africa affiliation but removing**  **Asia, SA and Africa (UR)** | |
| --- | --- | --- | --- | --- | --- | --- | --- | --- |
|  | **(1)** | **(2)** | **(3)** | **(4)** | **(5)** | **(6)** | **(7)** | **(8)** |
|  | **Omission from**  **authorship** | **Omission from acknowledgements** | **Omission from**  **authorship** | **Omission from acknowledgements** | **Omission from**  **authorship** | **Omission from acknowledgements** | **Omission from**  **authorship** | **Omission from acknowledgements** |
|  |  |  |  |  |  |  |  |  |
| Female | 0.97 | 1.01 | 0.96 | 1.02 | 0.96 | 1.01 | 0.96 | 1.02 |
|  | (0.04) | (0.05) | (0.04) | (0.05) | (0.04) | (0.05) | (0.04) | (0.05) |
| Non-White | 0.97 | 1.17*** | 1.01 | 1.15** | - | - | 1.02 | 1.15** |
|  | (0.05) | (0.07) | (0.05) | (0.07) |  |  | (0.05) | (0.07) |
| **Asia, SA and Africa affiliation** | **-** | **-** | **0.83**** | **1.06** | **0.83**** | **1.13** | **0.93** | **1.05** |
|  |  |  | **(0.07)** | **(0.11)** | **(0.06)** | **(0.11)** | **(0.05)** | **(0.08)** |
| Asia, SA and Africa (UR) | 1.07 | 1.01 | 1.18** | 0.99 | 1.18** | 1.00 | - | - |
|  | (0.06) | (0.07) | (0.08) | (0.09) | (0.08) | (0.09) |  |  |
| Observations | 7,752 | 7,752 | 7,559 | 7,559 | 7,559 | 7,559 | 7,559 | 7,559 |

Note: All other control variables were included and not reported here. Full Table reported in the Appendix to the response letter.

It is important to note that this new variable, “Asia, South America and Africa affiliation”, and the existing variable “Asia, South America and Africa (UR)”, are strongly positively correlated (corr=0.72). Given this high correlation, their coefficients are challenging to interpret. Note, for example, that the coefficient on “Asia, South America and Africa affiliation” is statistically significant in predicting stage 1 (Omission from authorship) in columns 3 and 5, but this changes when we remove the “Asia, South America and Africa (UR)” variable as reported in column 7.

Given the high correlations and the reduction in sample size that would result from introducing the “Asia, South America and Africa affiliation” variable, we have not included it in the main model. However, we now report this additional finding as a further analysis (see Table E in S2 File).

8. At the same time, the heterogeneity analyses about the race/gender X team characteristics is not well motivated and seems to distract from the key findings. If you can drop this, or limit to the Supplement, it might be better.

**Response:** We agree that these checks were somewhat distracting in the main text. We have now moved all further analysis of team characteristics, rather than the characteristics of the focal individuals, to Table D in the S2 File, alongside the corresponding text. We have also removed the corresponding figure (originally named Figure 2).

9. Is it possible to do an analysis of just the discerning omissions? Yes, the Ns get small, but this might be informative. But, showing wholesale exclusion versus discerning exclusion can address, for example, your conjectures about UR's not knowing norms versus URs discriminating against certain types of authors.

**Response:** We interpreted this comment as a request to distinguish between reviews that omitted all prior contributors (“wholesale omissions”) and those that selectively omitted some contributors (“discerning omissions”). In the Results section, we report that 88% of omissions occurred in reviews that failed to mention any prior contributors, whereas only 12% involved selective exclusion.

We attempted to re-estimate the model after recoding “Omission from the acknowledgements” as 1 only for the 72 individuals who were selectively omitted, and 0 otherwise. However, because these treated cases represent less than 1% of the full sample, several control variables perfectly predicted the outcome, leading to a substantial loss of observations (approximately 40%) in the second stage. Given the instability of the estimates under this operationalisation, we instead conducted two-sample tests of proportions to compare female and non-White contributors across “wholesale” and “discerning” omission cases. As reported in the table below, there is no significant difference in the proportion of females. Non-White individuals, however, are more represented in the “wholesale omissions” group, which refers to cases where no prior contributors were acknowledged at all.

**Two-sample test of proportions.**

|  | **“Wholesale**  **omissions”**  **n=549** | **“Discerning omissions”**  **n=72** | **Difference** | **Pr(Z < z)** |
| --- | --- | --- | --- | --- |
| Female | 0.45 | 0.53 | -0.07 | 0.12 |
| Non-White | 0.26 | 0.15 | 0.11 | 0.02 |

This pattern confirms our intuition that the observed disparities we observe for non-White contributors are driven more by weak or inconsistent acknowledgement practices (i.e., failures to credit anyone, “wholesale”) than by deliberate, selective exclusion of specific individuals from this group. We have added a new paragraph in the “Distribution of contributors omitted from the acknowledgements” section summarising this distinction between wholesale and discerning omissions. We also report the results of the two-sample tests in a new table, Table F in the S2 File.

10. At the same time, it is an unusual setting, and so you need to strongly acknowledge the limitations on generalizability (for example, see Jabbehdari and Walsh, 2017, STHV, for field differences in non-author collaborators).

**Response:** Thank you. We agree that the uniqueness of the Cochrane setting somewhat constrains the generalisability of our findings. We have now expanded the discussion of this limitation, suggesting that our setting is unique in at least three dimensions: the subject field (healthcare), the type of studies under investigation (systematic reviews), and the guidance and format of acknowledgements (e.g., explicit guidance.

We also read with interest the paper you recommended, and we now cite it in our discussion of generalisability. Following the Editor’s request, we now also discuss in the “Related Work” section of the paper how acknowledgement practices vary by discipline, which further sets the scene for these arguments.

Many thanks for your helpful comments.

**Reviewer #2:**

Opening comment. In this study, the authors use a corpus of approximately 2,000 Cochrane systematic reviews in medicine to investigate fairness in the attribution of credit in science, focusing in particular on the relationship between acknowledgments and formal authorship. They examine the factors that lead to omission of credit by analyzing the racial and gender characteristics of omitted contributors, and they show that geographical disparities—rather than explicit biases against specific communities—affect the likelihood of omission. I particularly appreciate the choice of the Cochrane corpus: the specific authorship and acknowledgment guidelines adopted by Cochrane enable to unpack the relationship between attribution and contribution in a way that is not possible with standard scientific publications. I also commend the substantial manual work involved in matching acknowledgees with authors and resolving ambiguities, which ensures the high quality of the dataset. I also value the authors’ attempt to measure the contribution of acknowledgees through keyword analysis. Furthermore, the discussion is broad and engaging, situating the study’s findings within the broader evolution of science toward more collective practices (e.g., citizen science) and exploring the challenges of fairly recognizing contributions in these contexts. Finally, the statistical methods used are robust.

**Response:** Many thanks for your kind words. We are delighted that you share our enthusiasm towards leveraging Cochrane as a suitable setting.

Before proceeding to publication, the following minor issues should be addressed:

1. Streamline the literature review to reduce overlap with the introduction.

**Response:** Thank you for this comment. The Editor and Reviewer 3 encouraged us to expand the literature review to provide a fuller theoretical and empirical context. At the same time, this comment suggested reducing overlap with the introduction.

We attempted to reconcile these requests by restructuring the “Related Work” section and removing some text from the “Introduction” to minimise repetition and improve flow, while retaining the breadth of coverage needed to situate our study within the relevant literature.

2. Clarify how the authors’ profiles were retrieved from Scopus: was this done manually or via the Scopus API?

**Response:** We now specify that all bibliometric data were retrieved using the Scopus Application Programming Interface (API). This clarification appears in the “Sample construction” subsection of the “Methods”.

3. Page 9: it is unclear whether the race attribution algorithm was independently validated by manually checking a sample of authors. Is the success rate of below 75% mentioned in the text the result of this validation?

**Response:** Thank you for allowing us to elaborate on this point. For each individual, the Ethnicolr algorithm returns the probability that their name is of Asian, Hispanic, Black, or White origin. It also returns the category with the highest probability as a categorical variable. Here is an example of the algorithm’s output:

**Ethnic classifier algorithm – example output.**

| **last_name** | **first_name** | **asian** | **hispanic** | **nh_black** | **nh_white** | **race** |
| --- | --- | --- | --- | --- | --- | --- |
| Kettle | Christine | 0.004106 | 0.018471 | 0.031101 | 0.946321 | nh_white |

We used the following decision rule: (1) if the individual was classified into a category and that category had more than 75% probability, we accepted the algorithm classification; (2) otherwise, we ran a web search to identify race.

The 75% threshold was chosen based on the average confidence of the prediction package in the sample. As expected, the algorithm predicts “White” names more confidently than “Asian, Hispanic, and Black” names. Specifically, in our sample, the average probability that a provided name belongs to a specific racial group is 85% for names classified as White and 76% for names classified as Non-White.

We believe this is a conservatively high threshold, compared to prior work. For example, Hofstra et al. (2020), who also utilise the Ethnicolr algorithm trained on Florida Voter Registration data in their checks, label a name as Hispanic if the probability returned by Ethnicolr exceeds 60%.

We have expanded the description of this process in the “Race” subsection. As suggested by Reviewer 3, we have also clarified, as early as the introduction, that this name-based ethnic classifier’s algorithm has imperfect accuracy (estimated at 83%).

Reference

Hofstra, B., Kulkarni, V. V., Munoz-Najar Galvez, S., He, B., Jurafsky, D., & McFarland, D. A. (2020). The diversity–innovation paradox in science. Proceedings of the National Academy of Sciences, 117(17), 9284-9291.

4. Specify how the variable Institutional Status was modeled: does it refer to the university’s position in the Times Higher Education ranking? How were cases handled where authors had multiple affiliations?

**Response:** Thank you for prompting us to clarify on this point. We assign an institutional affiliation based on a text search within the affiliation text. Specifically, we searched for all universities in the Times Higher Education (THE) ranking and created a dichotomous variable indicating whether each university was mentioned in the affiliation text.

For ease of interpretation (to ensure that institutions with higher ranks are classified as having higher institutional status), we then perform a linear transformation on the rank (400-x), so that the highest-ranked institutions have a higher value. For example, for the University of Oxford, which was ranked third, we set a value of 397:

**Example Institutional status variable.**

| **Rank** | **Institutional status** | **University** |
| --- | --- | --- |
| 3 | 397 | [University of Oxford](https://www.timeshighereducation.com/world-university-rankings/university-oxford) |
| 4 | 396 | [Stanford University](https://www.timeshighereducation.com/world-university-rankings/stanford-university) |
| 5 | 395 | [University of Cambridge](https://www.timeshighereducation.com/world-university-rankings/university-cambridge) |

In the text, we now report: “For ease of interpretation, we performed a linear transformation on the rank (400-x), so that the top-ranked institutions are classified as having higher institutional status. For example, for the University of Oxford, which was ranked third, we set a value of 397. For the University of Toronto, which was ranked twentieth, we set a value of 380.”

Thank you for enquiring about multiple affiliations. We had originally searched within the affiliation text for universities in the Times Higher Education list alone. We have now improved our algorithm to search for any university listed in the affiliation text, allowing us to examine how often authors list more than one university affiliation (even if these universities are not in the Times Higher Education ranking). These checks indicated that fewer than 1% of records had two or more affiliations, suggesting that single affiliations predominate.

We then manually reviewed cases with multiple identifiable institutions and, following standard academic convention, retained the first-listed university as the primary affiliation. So for example, for the affiliation text “*West China School of Public Health, West China Hospital, Sichuan University, Chengdu, China, zhenmiliu@scu.edu.cn / Division of Nursing, Midwifery and Social Work, School of Health Sciences, Faculty of Biology, Medicine and Health, University of Manchester, Manchester Academic Health Science Centre, Manchester, UK*” we identified two universities: (1) Sichuan University and (2) University of Manchester. To calculate the final rank, we retained Sichuan University’s rank, which is listed first.

We now use this improved operationalisation of “Institutional status” in all our models and recalculated all coefficients. The results are largely unaffected.

5. Table 1: clarify whether the reported statistics for the variables marked with “a” are log-transformed values or raw values before transformation.

**Response:** Thank you for suggesting this clarification. Our note now reads: “(a) raw values are reported; however, these variables were log-transformed prior to analysis”.

6. On page 21, among the studies applying network analysis to acknowledgments data, it would be appropriate to mention [DOI: 10.1007/s11229-022-03515-2].

**Response:** Thank you for bringing this interesting study by Petrovich (2022) to our attention. We have read it with interest and now cite it in the context of acknowledgement-based networks.

Thank you for your constructive comments.

**Reviewer #3:**

Opening comment. This study explores whether acknowledgements in academic publications omit deserving contributors and examines the heterogeneity of these omissions by gender and race. It raises interesting questions.

**Response:** We are glad you found the study raises interesting questions and are grateful for your helpful comments.

I would suggest clarifying the following points to enhance the manuscript's clarity.

1. First, the paper needs to improve its formatting for better readability, ensuring that both paragraphs are aligned.

**Response:** Thank you for this suggestion to improve the manuscript’s readability. We interpreted this comment to mean that the first line in each paragraph should be indented and accordingly indented all first lines. We remain prepared to make any further adjustments where needed.

2. The authors need to clarify the significance of their findings. What can be done by discovering the differences in acknowledgements related to race and gender?

**Response:** We appreciate this prompt to articulate practical implications. We have substantially expanded on the significance of the findings and their impact on practice in the “Discussion”, creating a separate subsection titled “Implications for practice”. This section translates our findings into concrete actions, including editorial guidance, author checklists, and training, and puts forward suggestions on how organisations, such as Cochrane and publishers, can strengthen fair acknowledgement practices.

Introduction

3. The authors should clarify how they determine the racial and gender identities of contributors, as well as the accuracy of the tool they use, in the introduction.

**Response:** Thank you, we have now amended the Introduction text to clarify that to study disparities, we use a name-based gender classifier, Gender-API and a name-based race classifier, Ethnicolr.

We elaborate on the important limitations of these approaches and their imperfect accuracy. In particular, we refer to recent evaluations that report ≈96% accuracy for Gender-API (see a recent study from PLOS Digital Health; VanHelene et al., 2024) and ≈83% for Ethnicolr (https://ethnicolr.readthedocs.io/ethnicolr.html).

Reference:

VanHelene, A. D., Khatri, I., Hilton, C. B., Mishra, S., Gamsiz Uzun, E. D., & Warner, J. L. (2024). Inferring gender from first names: Comparing the accuracy of Genderize, Gender-API, and the gender R package on authors of diverse nationality. PLOS Digital Health, 3: e0000456.

4. Emphasizing the contributions of this work is crucial; while the authors present observations and data, they should highlight the broader implications of these findings

**Response:** We agree that more details on the implications could have been anticipated in the paper’s introduction. We have strengthened the final paragraph of the Introduction to articulate the paper’s broader contribution: connecting credit attribution to the moral economy of science and demonstrating that disparities persist even under explicit acknowledgement guidance. As elaborated in our response to comment 2, we have also expanded on the implications for practice in the paper’s discussion.

5. I suggest to summarizing the research questions at the end of the introduction may also enhance clarity.

**Response:** Thank you. We have now amended the Introduction to include our research question.

Related work

6. The related work section needs to be more detailed and multi-dimensional. I suggest adding subheadings to enhance logical flow and coherence. It would also be beneficial to include a section on "The Impact of Race and Gender on Acknowledgement Practices." Additionally, more references should be incorporated regarding studies that use names to identify gender or race. Also, find related research that using Cochrane dataset.

**Response:** Thank you for this helpful comment. We have substantially expanded the “Related Work” section following your advice, as well as the recommendations put forward by the Editor. We now elaborate on how acknowledgements follow largely uncodified norms and highlight the nuanced nature of acknowledgement texts. We also engage with research showing disciplinary variation in acknowledgement practices. Following your advice, we also created a section on “The Impact of Gender and Race on Acknowledgement Practices”.

We believe the conceptual framework is stronger as a result of these changes. As Reviewer 2 requested that we streamline the literature review, we aimed to provide sufficient breadth while maintaining a concise text. We remain ready to make more modifications if deemed helpful.

Regarding your second point, we have now included references to recent studies that use the same gender and race classifier as we do in the Methods section. For Gender-API, we add that this tool has been used in previous studies to allocate binary gender (e.g., Pratschke et al. 2025). For Ethnicolr, we now mention that this approach has been used in other recent studies to categorise racial/ethnic categories of scientists (Hofstra et al., 2020; Morgan et al., 2022).

Finally, Cochrane’s ratings have been used among others in two papers stemming from the first author’s PhD research projects on (1) examining antecedents of selective reporting (Salandra, 2018) and (2) investigating whether Cochrane’s ratings help shift scientists towards better research (Salandra, Criscuolo, & Salter, 2021). Both studies use databases distinct from the one used in this submission and ask questions unrelated to the attribution of credit or acknowledgements. We decided to limit ourselves to adding only one of these references (Salandra, 2018) to the manuscript, to avoid excessive self-citation.

References:

Hofstra, B., Kulkarni, V. V., Munoz-Najar Galvez, S., He, B., Jurafsky, D., & McFarland, D. A. (2020). The diversity–innovation paradox in science. Proceedings of the National Academy of Sciences, 117: 9284-9291.

Morgan, A. C., LaBerge, N., Larremore, D. B., Galesic, M., Brand, J. E., & Clauset, A. (2022). Socioeconomic roots of academic faculty. Nature human behaviour, 6: 1625-1633.

Pratschke, K., Bristow, P., Paczesna, A., Parakh, I., MacKay, J. R., Mackay, F., & Blacklock, K. (2025). Marked gender inequity in the invited speakers at the European College of Veterinary Surgeons annual scientific congress 2012–2022. PLoS One, 20: e0329147.

Salandra R. (2018). Knowledge dissemination in clinical trials: exploring influences of institutional support and type of innovation on selective reporting. Research Policy, 47: 1215-1228.

Salandra R., Criscuolo P., & Salter A. (2021). Directing scientists away from potentially biased publications: The role of systematic reviews in health care. Research Policy, 50: 104130.

Method

7. Create a framework figure to illustrate the methodological approach, aiding comprehension for readers.

**Response:** We agree that a visual summary can aid comprehension for a broader multidisciplinary readership. We have therefore added a new figure illustrating the two-stage analytical framework. The figure summarises how the selection equation (“Omission from authorship”) and the outcome equation (“Omission from the acknowledgements”) are linked through the Inverse Mills Ratio, clarifying the sequential structure of the Heckman selection model. The figure and its description have been added to the S1 File (Figure C).

8. Why was the sample date created using reviews updated as of June 2019?

**Response:** Thank you for prompting us to clarify on this point. This reflects the time period during which our data collection was conducted. We have no particular reasons to believe that the underlying authorship and acknowledgement structures we analyse would be substantially different in the absence of this temporal cut-off.

First, we are not aware of any changes in Cochrane guidance or training regarding acknowledgements in recent years. Second, Cochrane has maintained their policy of regularly updating their reviews. Finally, as we estimate that the guidance has been well known within the Cochrane network at least since 2014, our time period should be sufficiently long to capture any delays in its uptake. Nevertheless, in our limitations, we now bring attention to this temporal cut-off.

9. There are 2,091 corresponding to 8,267 non-unique authors, but the same author name may appear in multiple reviews, representing different individuals. How should the issue of authors with the same name be addressed?

**Response:** We appreciate this observation. To minimise the risk of conflating individuals with the same name, we relied on the unique author identifiers provided by Elsevier’s Scopus database. Each author’s Scopus ID distinguishes individuals with the same name and links their publication histories unambiguously. We now clarify this in the “Sample Construction” section.

10. A flowchart would be beneficial to visually represent the methodological approach and decision-making process. Please include one.

**Response:** Thank you. In addition to Figure C, which illustrates our methodological framework that relies on the Heckman selection model, we have also included Figure B in File S1. This figure presents a flowchart of the data construction process, outlining each step from the identification of updated Cochrane reviews to the creation of the final analytical dataset, including the application of inclusion and exclusion criteria.

We hope we have interpreted your request correctly, but we remain prepared to make any further adjustments as needed.

Result

11. How to deal with the problem of data imbalance. Asian is 0.17; White is 0.77.

**Response:** Thank you. We agree that dealing with a highly imbalanced sample requires careful consideration. The imbalance inevitably reflects an imbalanced pool of Cochrane authors; however, it is also important to acknowledge that it may be exacerbated by our sampling method. In particular, from our original sample of 8,267 non-unique authors, we removed 515 authors for whom some information was not available (e.g., they were not retrievable in Scopus). Two-sample tests of proportions also show that ‘Non-White’ individuals are more prevalent among the 515 excluded and the 7,752 included records, with the difference in proportion being statistically significant (0.34 vs 0.23); therefore, we might be underestimating the prevalence of non-White authors. However, two-sample proportion tests show that ‘targeted’ omissions were equally frequent in both groups, indicating that the imbalance does not bias our estimates.

To correct for potential underrepresentation of the non-White group, in robustness checks, we employed the pweight=weights specification in STATA to assign probability weights to the non-White group. The weight was set to the inverse of the probability of a non-White individual being selected into the sample [weights = 0.235/.228 if Non-White ==1]. Our key finding, that non-White contributors were more likely to be omitted from the acknowledgements, remained robust with the inclusion of these weights.

Moreover, we note that this imbalance makes it more difficult to detect a significant association between author race and the omission from the acknowledgements. Because our sample includes a much larger proportion of White authors than non-White authors, the variance of our key independent variable (i.e. the “Non-White” dummy) is limited, which reduces statistical power. Therefore, the fact that we observe a statistically significant coefficient on “Non-White” despite the imbalance suggests that the underlying relationship is likely robust. In other words, a statistically significant effect, notwithstanding the imbalance, strengthens our confidence that the effect we observe is real and not spurious.

Thank you for prompting us to reflect on this imbalance. We have now adjusted the text to elaborate on the implications for our estimates.

12. The authors should avoid grouping multiple parts of a figure (e.g., Figure 1 A, B, C) into one and instead map distinct titles to each sub-figure for clarity.

**Response:** We fully agree with this suggestion. We have now split this figure into three separate ones: Figure 1, Figure 2, and Figure 3.

13. The discussion section can also include subheadings.

**Response:** We have now added two new subheadings, “Implications for research” and “Implications for practice”.

Many thanks for your helpful recommendations and detailed comments.

**Additional requirements raised by the editorial office:**

1. Please ensure that your manuscript meets PLOS ONE's style requirements, including those for file naming. The PLOS ONE style templates can be found at[https://journals.plos.org/plosone/s/file?id=wjVg/PLOSOne_formatting_sample_main_body.pdf](https://eur01.safelinks.protection.outlook.com/?url=https%3A%2F%2Fjournals.plos.org%2Fplosone%2Fs%2Ffile%3Fid%3DwjVg%2FPLOSOne_formatting_sample_main_body.pdf&data=05%7C02%7Crs2406%40bath.ac.uk%7C1a9d59720b454e2ee38b08ddfa76ed98%7C377e3d224ea1422db0ad8fcc89406b9e%7C0%7C0%7C638942111182294059%7CUnknown%7CTWFpbGZsb3d8eyJFbXB0eU1hcGkiOnRydWUsIlYiOiIwLjAuMDAwMCIsIlAiOiJXaW4zMiIsIkFOIjoiTWFpbCIsIldUIjoyfQ%3D%3D%7C0%7C%7C%7C&sdata=IMY%2FeKq0JkZ1p6yjZkTudE5lnjKZNLgDXT6MQJaWMbA%3D&reserved=0) and[https://journals.plos.org/plosone/s/file?id=ba62/PLOSOne_formatting_sample_title_authors_affiliations.pdf](https://eur01.safelinks.protection.outlook.com/?url=https%3A%2F%2Fjournals.plos.org%2Fplosone%2Fs%2Ffile%3Fid%3Dba62%2FPLOSOne_formatting_sample_title_authors_affiliations.pdf&data=05%7C02%7Crs2406%40bath.ac.uk%7C1a9d59720b454e2ee38b08ddfa76ed98%7C377e3d224ea1422db0ad8fcc89406b9e%7C0%7C0%7C638942111182305229%7CUnknown%7CTWFpbGZsb3d8eyJFbXB0eU1hcGkiOnRydWUsIlYiOiIwLjAuMDAwMCIsIlAiOiJXaW4zMiIsIkFOIjoiTWFpbCIsIldUIjoyfQ%3D%3D%7C0%7C%7C%7C&sdata=FAfgWhc7CRp8XBkiV5owJ%2Bm%2Bdwza%2BlUDey0LR3PgyzI%3D&reserved=0)

**Response:** Thank you. We reviewed the manuscript's formatting and the file-naming conventions. We have also reviewed the affiliations, removing all street addresses, postcodes and abbreviations.

2. Please include your full ethics statement in the ‘Methods’ section of your manuscript file. In your statement, please include the full name of the IRB or ethics committee who approved or waived your study, as well as whether or not you obtained informed written or verbal consent. If consent was waived for your study, please include this information in your statement as well.

**Response:** We have now added the following sentence in the Methods section of the manuscript: “This project obtained a favourable ethics opinion from the University of Bath Data & Digital Science Research Ethics Committee (Ref: 1543-1206). Informed consent was not obtained as the project used secondary data that was available in the public domain.”

3. We note that you have indicated that there are restrictions to data sharing for this study. PLOS only allows data to be available upon request if there are legal or ethical restrictions on sharing data publicly. For more information on unacceptable data access restrictions, please see [http://journals.plos.org/plosone/s/data-availability#loc-unacceptable-data-access-restrictions](https://eur01.safelinks.protection.outlook.com/?url=http%3A%2F%2Fjournals.plos.org%2Fplosone%2Fs%2Fdata-availability%23loc-unacceptable-data-access-restrictions&data=05%7C02%7Crs2406%40bath.ac.uk%7C1a9d59720b454e2ee38b08ddfa76ed98%7C377e3d224ea1422db0ad8fcc89406b9e%7C0%7C0%7C638942111182315171%7CUnknown%7CTWFpbGZsb3d8eyJFbXB0eU1hcGkiOnRydWUsIlYiOiIwLjAuMDAwMCIsIlAiOiJXaW4zMiIsIkFOIjoiTWFpbCIsIldUIjoyfQ%3D%3D%7C0%7C%7C%7C&sdata=b1BtWs4SystTftuNVFwN0kGqaHS0Qf8zDE2gTd%2BqB1g%3D&reserved=0). Before we proceed with your manuscript, please address the following prompts:
   b) If there are no restrictions, please upload the minimal anonymized data set necessary to replicate your study findings to a stable, public repository and provide us with the relevant URLs, DOIs, or accession numbers. For a list of recommended repositories, please see
   [https://journals.plos.org/plosone/s/recommended-repositories](https://eur01.safelinks.protection.outlook.com/?url=https%3A%2F%2Fjournals.plos.org%2Fplosone%2Fs%2Frecommended-repositories&data=05%7C02%7Crs2406%40bath.ac.uk%7C1a9d59720b454e2ee38b08ddfa76ed98%7C377e3d224ea1422db0ad8fcc89406b9e%7C0%7C0%7C638942111182325339%7CUnknown%7CTWFpbGZsb3d8eyJFbXB0eU1hcGkiOnRydWUsIlYiOiIwLjAuMDAwMCIsIlAiOiJXaW4zMiIsIkFOIjoiTWFpbCIsIldUIjoyfQ%3D%3D%7C0%7C%7C%7C&sdata=%2BkeZyhjqKJb0bwUvwIWu8ca1kZKW5Sr8hskFhBm3JCw%3D&reserved=0). You also have the option of uploading the data as Supporting Information files, but we would recommend depositing data directly to a data repository if possible. We will update your Data Availability statement on your behalf to reflect the information you provide.

**Response:** We have now created an anonymised version of our dataset, which is available on the University of Bath Research Data Archive.

The URL for this record is: <https://researchdata.bath.ac.uk/id/eprint/1609>.

Our Data Services recommended that the DOI (Digital Object Identifier) be used instead of the URL, once activated: <https://doi.org/10.15125/BATH-01609>.

The record has been marked as ready to publish, but the dataset will not be visible to others until we are prepared to make it public. If the reviewers need access to the dataset, the Data Services can set up a restricted-access preview for them.

4. Please include captions for your Supporting Information files at the end of your manuscript, and update any in-text citations to match accordingly. Please see our Supporting Information guidelines for more information: http://journals.plos.org/plosone/s/supporting-information.

**Response:** We have now renamed our supporting information files as S1_File (formerly S1 Figure) and S2_File (formerly S1 Table) and included captions at the end of the manuscript.

5. We note that there is identifying data in the Supporting Information file <S1 Figure.docx>. Due to the inclusion of these potentially identifying data, we have removed this file from your file inventory. Prior to sharing human research participant data, authors should consult with an ethics committee to ensure data are shared in accordance with participant consent and all applicable local laws. Data sharing should never compromise participant privacy. It is therefore not appropriate to publicly share personally identifiable data on human research participants.

**Response:** We consulted with our ethics committee on this matter. Please note that this supplemental information file has been renamed S1 File (previously named S1 Table). The original file included two figures:

a) The first figure reports bibliometric information from the Cochrane website. We agree that this includes some personal information, specifically individual names and surnames. However, it is publicly available, as it simply refers to the author’s names. As such, we believe there should be no concerns in sharing this information. We also obtained separate permission from Wiley and Sons (publisher of the Cochrane Library) to reuse this figure.
b) The second figure also reports information from the Cochrane website. This information is also publicly available. However, given the potentially sensitive nature of some of this information (for example, the identities of individuals acknowledged) and the fact that a reader could easily access it directly from the Cochrane website if needed, we decided to remove this figure from S1 File.

We hope this helps clarify the matter; however, we remain prepared to make any further adjustments as needed.

**Appendix to the response letter**

**Appendix Table 1. Main results with the additional control “Asia, S. America and Africa affiliation”.**

|  | **Original model** | | **With additional control:**  **Asia, S. America and Africa affiliation** | | **With additional control:**  **Asia, S. America and Africa affiliation but removing non-White** | | **With additional control:**  **Asia, S. America and Africa affiliation but removing**  **Asia, South America and Africa (UR)** | |
| --- | --- | --- | --- | --- | --- | --- | --- | --- |
|  | **(1)** | **(2)** | **(3)** | **(4)** | **(5)** | **(6)** | **(7)** | **(8)** |
|  | **Omission from**  **authorship** | **Omission from acknowledgements** | **Omission from**  **authorship** | **Omission from acknowledgements** | **Omission from**  **authorship** | **Omission from acknowledgements** | **Omission from**  **authorship** | **Omission from acknowledgements** |
|  |  |  |  |  |  |  |  |  |
| Female | 0.97 | 1.01 | 0.96 | 1.02 | 0.96 | 1.01 | 0.96 | 1.02 |
|  | (0.04) | (0.05) | (0.04) | (0.05) | (0.04) | (0.05) | (0.04) | (0.05) |
| Non-White | 0.97 | 1.17*** | 1.01 | 1.15** |  |  | 1.02 | 1.15** |
|  | (0.05) | (0.07) | (0.05) | (0.07) |  |  | (0.05) | (0.07) |
| **Asia, South America and Africa affiliation** |  |  | 0.83** | 1.06 | 0.83** | 1.13 | 0.93 | 1.05 |
|  |  |  | (0.07) | (0.11) | (0.06) | (0.11) | (0.05) | (0.08) |
| Academic influence | 0.95*** | 1.01 | 0.95*** | 1.01 | 0.95*** | 1.00 | 0.95*** | 1.01 |
|  | (0.01) | (0.02) | (0.01) | (0.02) | (0.01) | (0.02) | (0.01) | (0.02) |
| First author | 0.44*** | 0.93 | 0.44*** | 0.91 | 0.44*** | 0.91 | 0.44*** | 0.91 |
|  | (0.02) | (0.08) | (0.02) | (0.08) | (0.02) | (0.08) | (0.02) | (0.08) |
| Last author | 0.78*** | 0.94 | 0.79*** | 0.94 | 0.79*** | 0.95 | 0.78*** | 0.94 |
|  | (0.03) | (0.05) | (0.03) | (0.05) | (0.03) | (0.06) | (0.03) | (0.05) |
| Institutional status | 1.00 | 1.00 | 1.00 | 1.00 | 1.00 | 1.00 | 1.00 | 1.00 |
|  | (0.00) | (0.00) | (0.00) | (0.00) | (0.00) | (0.00) | (0.00) | (0.00) |
| Research protocol (OR) | 0.73** | 1.13 | 0.68** | 1.09 | 0.68** | 1.10 | 0.68** | 1.09 |
|  | (0.10) | (0.21) | (0.11) | (0.22) | (0.11) | (0.23) | (0.11) | (0.22) |
| Institutional status team lead (UR) | 1.00*** | 1.00** | 1.00*** | 1.00** | 1.00*** | 1.00** | 1.00*** | 1.00** |
|  | (0.00) | (0.00) | (0.00) | (0.00) | (0.00) | (0.00) | (0.00) | (0.00) |
| External funding (UR) | 1.09** | 1.05 | 1.10** | 1.05 | 1.10** | 1.04 | 1.10** | 1.05 |
|  | (0.05) | (0.06) | (0.05) | (0.06) | (0.05) | (0.06) | (0.05) | (0.06) |
| Length acknowledgements | 1.16*** | 0.74*** | 1.15*** | 0.74*** | 1.15*** | 0.74*** | 1.15*** | 0.74*** |
|  | (0.03) | (0.02) | (0.03) | (0.02) | (0.03) | (0.02) | (0.03) | (0.02) |
| Authors count (UR) | 0.93*** | 1.07*** | 0.92*** | 1.07*** | 0.92*** | 1.07*** | 0.92*** | 1.07*** |
|  | (0.01) | (0.01) | (0.01) | (0.01) | (0.01) | (0.01) | (0.01) | (0.01) |
| Asia, South America and Africa (UR) | 1.07 | 1.01 | 1.18** | 0.99 | 1.18** | 1.00 |  |  |
|  | (0.06) | (0.07) | (0.08) | (0.09) | (0.08) | (0.09) |  |  |
| Years difference | 1.09*** |  | 1.09*** |  | 1.09*** |  | 1.09*** |  |
|  | (0.01) |  | (0.01) |  | (0.01) |  | (0.01) |  |
| No Recent publication | 2.18*** |  | 2.17*** |  | 2.17*** |  | 2.16*** |  |
|  | (0.11) |  | (0.11) |  | (0.11) |  | (0.11) |  |
| invmills |  | 0.36*** |  | 0.36*** |  | 0.37*** |  | 0.36*** |
|  |  | (0.03) |  | (0.03) |  | (0.03) |  | (0.03) |
| Constant | 0.19*** | 4.09*** | 0.17*** | 4.67*** | 0.17*** | 4.67*** | 0.17*** | 4.62*** |
|  | (0.06) | (1.44) | (0.07) | (2.01) | (0.07) | (2.01) | (0.07) | (1.99) |
|  |  |  |  |  |  |  |  |  |
| Observations | 7,752 | 7,752 | 7,559 | 7,559 | 7,559 | 7,559 | 7,559 | 7,559 |
